# Gut Microbiome as a Potential Biomarker in Fish: Dietary Exposure to Petroleum Hydrocarbons and Metals, Metabolic Functions and Cytokine Expression in Juvenile *Lates calcarifer*

**DOI:** 10.3389/fmicb.2022.827371

**Published:** 2022-07-22

**Authors:** Francis Spilsbury, Md Javed Foysal, Alfred Tay, Marthe Monique Gagnon

**Affiliations:** ^1^School of Molecular and Life Sciences, Curtin University, Bentley, WA, Australia; ^2^Department of Genetic Engineering and Biotechnology, Shahjalal University of Science and Technology, Sylhet, Bangladesh; ^3^Helicobacter Research Laboratory, The Marshall Centre, School of Biomedical Sciences, The University of Western Australia, Perth, WA, Australia

**Keywords:** barramundi, ecotoxicology, metals, crude oil, gut microbiome, cytokines, bioinformatics, biomarkers

## Abstract

The gut microbiome of fish contains core taxa whose relative abundances are modulated in response to diet, environmental factors, and exposure to toxicogenic chemicals, influencing the health of the host fish. Recent advances in genomics and metabolomics have suggested the potential of microbiome analysis as a biomarker for exposure to toxicogenic compounds. In this 35-day laboratory study, 16S RNA sequencing and multivariate analysis were used to explore changes in the gut microbiome of juvenile *Lates calcarifer* exposed to dietary sub-lethal doses of three metals: vanadium (20 mg/kg), nickel (480 mg/kg), and iron (470 mg/kg), and to two oils: bunker C heavy fuel oil (HFO) (1% w/w) and Montara, a typical Australian medium crude oil (ACO) (1% w/w). Diversity of the gut microbiome was significantly reduced compared to negative controls in fish exposed to metals, but not petroleum hydrocarbons. The core taxa in the microbiome of negative control fish comprised phyla Proteobacteria (62%), Firmicutes (7%), Planctomycetes (3%), Actinobacteria (2%), Bacteroidetes (1%), and others (25%). Differences in the relative abundances of bacterial phyla of metal-exposed fish were pronounced, with the microbiome of Ni-, V-, and Fe-exposed fish dominated by Proteobacteria (81%), Firmicutes (68%), and Bacteroidetes (48%), respectively. The genus *Photobacterium* was enriched proportionally to the concentration of polycyclic aromatic hydrocarbons (PAHs) in oil-exposed fish. The probiotic lactic acid bacterium *Lactobacillus* was significantly reduced in the microbiota of fish exposed to metals. Transcription of cytokines IL-1, IL-10, and TNF-a was significantly upregulated in fish exposed to metals but unchanged in oil-exposed fish compared to negative controls. However, IL-7 was significantly downregulated in fish exposed to V, Ni, Fe, and HFOs. Fish gut microbiome exhibits distinctive changes in response to specific toxicants and shows potential for use as biomarkers of exposure to V, Ni, Fe, and to PAHs present in crude oil.

## Graphical Abstract

Dietary exposure to petroleum hydrocarbons and metals leads to changes in the gut microbiome and cytokine expression in *Lates calcarifer*. 
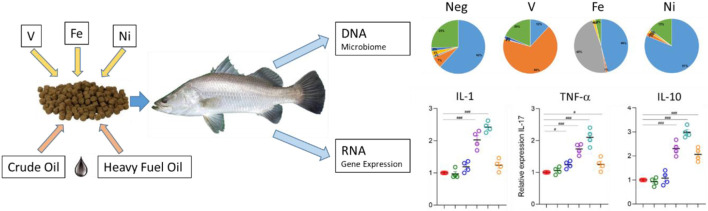

## HIGHLIGHTS

- Fish exposed to dietary metals and crude oils exhibit changes in the gut microbiome.
- *Photobacterium* is identified as a potential biomarker genus for high m.w. polycyclic aromatic hydrocarbon (PAH) exposure.
- Dietary V, Fe and Ni enrich phyla Firmicutes, Bacteroidetes and Proteobacteria respectively.
- Microbiome diversity is reduced by dietary metals, but not by petroleum hydrocarbons.
- IL-1, IL-10, and TNF-a expression increases after dietary exposure to metals and heavy fuel oil (HFO).

## Introduction

The microbiome of the gastrointestinal tract in fish plays an important role in maintaining the overall health of fish (Hoseinifar et al., 2019), including bi-directional biochemical interactions that influence their immune system (Gómez and Balcázar, 2008; Xia et al., 2014; Adamovsky et al., 2018). The “typical” makeup of the fish gut microbiome is composed of core taxa of bacteria predominantly of the phyla Proteobacteria, Firmicutes, Actinobacteria, Fusobacteria, and Bacteroidetes (Cahill, 1990; Gómez and Balcázar, 2008; Roeselers et al., 2011; Ghanbari et al., 2015; Adamovsky et al., 2018). The relative abundance of genera present in the gut microbiome varies greatly between species of fish (Givens et al., 2015; Egerton et al., 2018; Nikouli et al., 2021) and between individuals within a species (Burke et al., 2011).

The trophic level, and thereby diet, is the predominant factor influencing the relative abundances of phyla present in the gut microbiome of fish (Estruch et al., 2015; Egerton et al., 2018; Talwar et al., 2018; reviewed by Legrand et al., 2020). *Lates calcarifer* (barramundi or Asian seabass) is a popular sports fish and is a common aquaculture species farmed throughout Asia. Dietary studies have established the relative prevalence of taxa in the microbiome of *L. calcarifer* (Xia et al., 2014; Gupta et al., 2020), which is generally similar to that found in other comparable species of carnivorous fish (Egerton et al., 2018). Changes in diet result in a change in the relative abundance of dominant genera in the gut microbiome of *L. calcarifer* (Gupta et al., 2020) and other fish species (Xia et al., 2014; Estruch et al., 2015; Ringø et al., 2016; Egerton et al., 2018).

Exposure to anthropogenic toxicants such as metals and petroleum hydrocarbons also alters the gut microbiome in fish, as illustrated by field studies following the Deepwater Horizon (DWH) spill (Brown-Peterson et al., 2015) and a riverine oil spill in Saskatchewan, Canada (DeBofsky et al., 2020). Laboratory studies have shown that red bream (*Pagrus major*) exposed to phenanthrene produces significant changes in gut microbiome (Hano et al., 2021). Similarly, benthic microbial communities exhibit a profile shift following exposure to mixtures of benzo(*a*)pyrene and fluorene (Kahla et al., 2021). Perhaps more ecologically relevant, exposing lined sole (*Achirus lineatus*) to water accommodated fractions (WAF) generated from crude oil from the DWH spill has similarly shown to produce significant changes in the relative abundances of bacterial genera of the gut microbiome (Améndola-Pimenta et al., 2020).

The gut microbiome plays a significant role in the overall metabolic outcomes of the host organism challenged by environmental toxicants. For example, bacterial metabolism assists the host fish in the detoxification of ammonia (Turner and Bucking, 2019). The gut microbiome of farmed *Scophthalmus maximus* (turbot) contains genes for heavy metal resistance and exhibits a functional emphasis of iron uptake and metabolism (Xing et al., 2013). Vanadium nitrogenase facilitates an alternative pathway for nitrogen fixation (Gustafsson, 2019), utilized by *Pseudomonas* and *Cyanobacteria* species, among others (Lyalkova and Yurkova, 1992; Pessoa et al., 2015). *Lactobacillus*, a lactic acid-producing bacterium used as a probiotic in aquaculture, is associated with improved resilience against bacterial and viral pathogens (He et al., 2017; Collins, 2019) and moderates the effects of lead (Giri et al., 2018) and cadmium (Zhai et al., 2017) exposure.

Crude oils are highly complex mixtures of compounds, which may enter food webs in the event of a spill (Buskey et al., 2016) and subsequently biomagnify in species of exposed fish to levels as high as 2.2% w/w (Sammarco et al., 2013). Persisting in the environment for several years post-release (Boehm et al., 2008), petroleum hydrocarbons are retained in the tissues of fish for months after exposure has ceased (Cravedi and Tulliez, 1986), and ecotoxicological biomarkers indicating continued exposure remain elevated months after an oil spill (Smeltz et al., 2017).

In contaminated environments, bacterial communities shift toward those resistant taxa that are able to metabolize or sequester toxicants. For example, microbial communities in oil-contaminated soils contain polycyclic aromatic hydrocarbon (PAH)-metabolizing bacteria (Zafra et al., 2014; Lee et al., 2018; Haritash, 2020), and bacterial communities in vanadium-contaminated soils were found to be dominated by Bacteroidetes, Proteobacteria, Actinobacteria, and Firmicutes (Zhang et al., 2018, 2019; Lu et al., 2019), all of which are core taxa found in abundance in the gut microbiome of many species of fish. It seems likely that the gut microbiome of fish exposed to toxicants such as PAHs or metals may become dominated by those taxa able to metabolize those contaminants, and thereby reduce the toxic burden on the host organism by co-metabolization.

Changes in the gut microbiome in response to toxicants may reduce the community complexity (DeBofsky et al., 2021) and alter the metabolic outcomes of the bacterial communities present (Adamovsky et al., 2018), and therefore could be used in ecotoxicological fingerprinting to identify classes of anthropogenic toxicants to which the organisms are exposed (Adamovsky et al., 2018; Walter et al., 2019).

In this study, we present the analysis of the gut microbiome of *L. calcarifer* exposed *via* diet to a bunker C heavy fuel oil (HFO), to a typical Australian medium crude oil (ACO), and to three mixtures of a selection of petroleum hydrocarbons enriched with sub-lethal doses of vanadium, nickel, and iron, respectively. Non-metric multidimensional scaling (nMDS) analysis was used to differentiate the microbiome community profiles of the various exposure groups, and comparative analyses of dominant phyla and genera were used alongside cytokine gene expression in the gut microbiome to ascertain the suitability of fish gut genomics as a potential ecotoxicological biomarker.

## Materials and Methods

### *In vivo* Fish Exposure and Sampling

All fish were handled in accordance with Curtin University animal ethics approval (number ARE2019/11).

Juvenile fish (*n* = 56; 10–15 cm in length; mean weight 85.0 g) were obtained from a local commercial hatchery. Following a 5-day acclimatization to 32 ppt saline conditions, the fish were placed in tanks containing 100 L of natural Indian Ocean seawater sourced from north of Perth, Australia, with four fish per tank. A static-renewal design was used with a 12-h light/dark interval. Water quality was maintained at 28 ± 2°C, 32 ± 4 ppt salinity, pH 7.6 ± 0.6, dissolved oxygen 5.0 mg/L, and total ammonia < 2.0 mg/L, assisted by Astro 2212 external canister biofilters with a flow rate of approximately 5 L/min and up to 50% daily water exchanges as required.

The fish were fed 2% body weight per day commercial fishmeal (Nova FF, Skretting Pty Ltd., Perth, WA, Australia), in line with similar exposure trials (e.g., Hellou and Leonard, 2004). Toxicant dosage rates were set to align with fish no observed effect concentrations (NOECs) to ensure fish survival throughout the trial. Due to a paucity of ecotoxicological data specifically for *L. calcarifer*, the sub-lethal dosage of metals and individual petroleum hydrocarbons was estimated using published NOEC data for mortality of other fish species (Hilton and Bettger, 1988; Ptashynski et al., 2002; Craig et al., 2009; U.S. Environmental Protection Agency, 2020). Dosage rates for crude oil were similar to those in other dietary exposure studies (Nahrgang et al., 2010; Vieweg et al., 2018).

Fish in the negative control group were fed unaltered fishmeal. Fish in the petroleum hydrocarbon test groups were fed fishmeal spiked with 1% w/w ACO, or fishmeal spiked with 1% w/w HFO. An additional three groups were fed fishmeal enriched with a small amount of a mixture of aromatic and saturated petroleum hydrocarbons (total petroleum hydrocarbons approximately 25 mg/kg) and 20 mg/kg vanadium (V), 470 mg/kg iron (Fe), or 480 mg/kg nickel (Ni). The detailed composition of fishmeal given to each treatment group is summarized in Table 1 and Supplementary Tables 1, 2.

**TABLE 1:** Toxicant additives in fish feed.^§^

	Compound	Neg control (mg/kg)	1% ACO fish feed (mg/kg)	1% HFO fish feed (mg/kg)	V-enriched fish feed (mg/kg)	Fe-enriched fish feed (mg/kg)	Ni-enriched fish feed (mg/kg)
Bicyclic aromatics	Naphthalene	<0.5	41.33	12.33	2.13	<0.1	<0.1
	2-methylnaphthalene	<0.5	116.67	37.33	<0.1	<0.1	<0.1
	1-methylnaphthalene	<0.5	57.33	21.33	<0.1	2.17	<0.1
	C2-alkylnaphthalenes	<0.5	136.67	52.33	<0.5	<0.5	2.47
	C3-alkylnaphthalenes	<0.5	85.00	44.33	<0.5	<0.5	<0.5
	C4-alkylnaphthalenes	<0.5	31.00	21.67	<0.5	<0.5	<0.5
	Dibenzothiophene	<0.5	3.87	3.43	0.87	<0.5	< 0.5
	Methyldibenzothiophenes	<0.5	7.17	11.67	<0.5	<0.5	<0.5
	C2-alkyldibenzothiophenes	<0.5	7.13	21.33	<0.5	<0.5	<0.5
	C3-alkyldibenzothiophenes	<0.5	3.70	18.33	<0.5	<0.5	<0.5
	Acenaphthylene	<0.5	<0.5	<0.5	<0.5	<0.5	<0.5
	Acenaphthene	<0.5	1.47	0.97	<0.5	<0.5	<0.5
	**Total Bicyclic aromatics**	**<0.5**	**491**	**245**	**3.00**	**2.17**	**2.47**
Tricyclic aromatics	Phenanthrene	<0.5	19.00	5.33	0.90	<0.5	<0.5
	Methylphenanthrenes	<0.5	34.67	17.00	<0.5	<0.5	<0.5
	C2-alkylphenanthrenes	<0.5	30.00	26.67	0.17	2.77	0.57
	C3-alkylphenanthrenes	<0.5	46.33	52.00	<0.5	<0.5	<0.5
	C4-alkylphenanthrenes	<0.5	6.43	11.67	0.63	0.60	<0.5
	Anthracene	<0.5	<0.5	0.70	<0.5	<0.5	<0.5
	**Total Tricyclic aromatics**	**<0.5**	**136**	**113**	**1.70**	**3.37**	**0.57**
Tetracyclic aromatics	Fluoranthene	<0.5	0.23	<0.5	<0.5	<0.5	<0.5
	Pyrene	<0.5	0.60	1.53	2.50	<0.5	<0.5
	Benzo(a)anthracene	<0.5	<0.5	1.13	<0.5	<0.5	<0.5
	Chrysene	<0.5	0.97	2.30	<0.5	<0.5	<0.5
	Methylpyrenes/fluoranthenes	<0.5	4.97	6.93	<0.5	<0.5	<0.5
	C2-alkylpyrenes/fluoranthenes	<0.5	4.77	13.00	<0.5	<0.5	<0.5
	C3-alkylpyrenes/fluoranthenes	<0.5	3.23	13.00	<0.5	<0.5	<0.5
	Methylchrysenes	<0.5	1.27	8.57	<0.5	<0.5	<0.5
	C2-alkylchrysenes	<0.5	1.67	14.67	0.87	<0.5	<0.5
	Methylindenopyrenes	<0.5	0.50	2.90	0.33	<0.5	<0.5
	C2-alkylindenopyrenes	<0.5	<0.5	2.70	<0.5	<0.5	<0.5
	Fluorene	<0.5	10.67	1.83	3.20	<0.5	<0.5
	**Total Tetracyclic aromatics**	**<0.5**	**28.9**	**68.6**	**6.90**	**<0.5**	**<0.5**
Pentacyclic armoatics	Benzo(b)fluoranthene	<0.5	<0.5	0.33	<0.5	<0.5	<0.5
	Benzo(k)fluoranthene	<0.5	<0.5	<0.5	<0.5	<0.5	<0.5
	Benzo(a)pyrene	<0.5	<0.5	1.20	<0.5	<0.5	<0.5
	Indeno(1,2,3-cd)pyrene	<0.5	<0.5	<0.5	<0.5	<0.5	<0.5
	Dibenzo(a,h)anthracene	<0.5	<0.5	<0.5	<0.5	<0.5	<0.5
	Benzo(g,h,i)perylene	<0.5	<0.5	<0.5	<0.5	<0.5	<0.5
	Methylbenzopyrenes	<0.5	<0.5	5.17	<0.5	<0.5	<0.5
	C2-alkylbenzopyrenes	<0.5	0.37	7.23	<0.5	<0.5	<0.5
	**Total Pentacyclic aromatics**	**<0.5**	**0.37**	**13.9**	**<0.5**	**<0.5**	**<0.5**
Metals	Sulfur	0	3.9	102	0	270	260
	Vanadium	0	0	0.15	19.4	0	0
	Iron	0	0	0.38	0	470	0
	Nickel	0	0	0.12	0	0	480
	Aluminum	0	0.31	0.15	0	0	0
	Tin	0	0.18	0.13	0	0	0

*Amounts reported are an average of triplicate analyses. “<0.1” or “<0.5” denotes no amounts detected above the respective limits of reporting. ^§^See also Supplementary Tables 1, 2.*

The fish were exposed for a total of 33 days, followed by a 2-day depuration period before euthanasia using the ike-jime technique (Davie and Kopf, 2006). The intestinal tract was removed and stripped using Teflon tweezers, and whole gut contents were collected in 2-mL cryovials that were immediately frozen in liquid nitrogen and then stored at –80°C until analysis.

An outline of study design is presented in Supplementary Figure 1.

### Polycyclic Aromatic Hydrocarbon and Metal Analysis of Fish Feed

#### Polycyclic Aromatic Hydrocarbons

Fish feeds used in the trial were analyzed for a suite of 38 PAHs in a commercial consultant laboratory (ChemCentre, Perth, WA, Australia) using standard published methods (Forth et al., 2017). Analyses were performed in triplicate.

Briefly, an internal standard was added to precisely weighed samples of fish feeds, and extraction was performed by sonication in acetone/dichloromethane, followed by chemical drying with sodium sulfate. Quantitation by GC-MS (SIM) was against a commercially available standard (AccuStandard, New Haven, CT, United States). The response factors of the respective parent PAH were used to quantitate alkylated PAHs (Forth et al., 2017).

#### Metals Analysis

Metals in crude oils were quantified by ICP-AES and ICP-MS in a commercial laboratory (TSW Analytical, Perth, WA, Australia). Analyses for a suite of 61 metals were performed in triplicate. Briefly, an accurately weighed sample of oil was digested in nitric acid repeatedly and then finally in a mixture of nitric and perchloric acid. Once taken to incipient dryness, the digestate was re-dissolved in nitric acid, hydrochloric acid, and high purity water. Quantification was performed against a commercially available standard (AccuTrace High Purity multi-element standards, Choice Analytical, Sydney, NSW, Australia).

### Microbiome Analysis

#### Collection and Processing of Samples

Water samples were collected before the start of the experiment. One set of freshly prepared feed samples was stored immediately at –80°C and collected in triplicate from each group after the trial. At the end of the trial, the intestinal content was taken from each fish for microbiome analysis: negative control (*n* = 12), ACO (*n* = 12), HFO (*n* = 12), V-enriched (*n* = 4), Fe-enriched (*n* = 8), and Ni-enriched (*n* = 8) diets. Concurrently, samples of feed (*n* = 4) were collected randomly from each of the six dietary test groups, and seawater samples (*n* = 6) were collected before the start of the trial from the marine water supply chain. The gut samples of fish were collected inside a biosafety cabinet. Precisely 200 mg of gut and feed samples with 100 μl of DEPC-treated water were homogenized using a tissue lyser (Qiagen, Hilden, Germany) with beads. The water samples (200 ml) were collected in sterile falcon tubes (50 ml ×4), followed by filtration in 0.2-μm polycarbonate filters with a Sentino Microbiology Pump (Pall Corporation, New York, United States). The filters were then cut into small pieces (∼1 mm) inside a biosafety cabinet and transferred into 2-ml bead tubes of the DNeasy PowerSoil Kit (Qiagen, Hilden, Germany).

#### DNA Extraction and PCR Amplification of 16S rRNA Gene

Bacterial DNA from 86 processed samples (56 gut, 24 feed, and 6 water) was extracted using a DNeasy PowerSoil Kit (Qiagen, Hilden, Germany) following the manufacturer’s instructions. The quantity and quality of DNA were checked using a NanoDrop Spectrophotometer 2000c (Thermo Fisher Scientific, Waltham, MA, United States). A final DNA concentration of 50 ng/μl was achieved by dilution. PCR amplification of V3-V4 hypervariable regions of bacteria was performed according to the Illumina 16S metagenomic sequencing protocol (Part # 15044223 Rev. B). A 50 μl of PCR master mix was prepared by mixing 2 μl of template DNA (50 ng/μl), 1 μl each of forward and reverse primers, 25 μl of Hot Start Taq 2X Master Mix (New England BioLab Inc., Ipswich, MA, United States), and 21 μl DEPC-treated water. A total of 35 cycles of amplification were performed in a S1000 Gradient Thermal Cycler (Bio-Rad Laboratories, Inc., Foster City, CA, United States). Bead purification, amplicon barcoding, and pooling were performed according to the Illumina 16S standard protocol (Part # 15044223 Rev. B). Sequencing was performed with Illumina MiSeq platforms (Illumina Inc., San Diego, CA, United States) using a MiSeq reagent kit (600 cycles, Part # MS-102-3003).

#### Processing of Illumina Reads

The initial quality of raw fastq sequences was checked by FastQC (Andrews, 2010), multiQC (Ewels et al., 2016), and Micca stat (Albanese et al., 2015). Trimming of low-quality reads and removal of adapter sequences were performed using BBduk (Bushnell, 2014) with the following parameters: qtrim = r, trimq = 20, ktrim = r, k = 23, mink = 11, hdist = 1, minlen = 200, tpe, tbo. The merging, filtering, de-duplicating (fastq-uniques), and picking of amplicon sequence variants (ASVs) was performed in a USEARCH pipeline by implementing UPARSE and UNOISE3 (Edgar, 2010, 2013, 2016a). The final set of ASVs was filtered for chimeras using UCHIME2 (Edgar, 2016b). UNOISE3 flow was used to map all the merge reads to a non-chimeric ASV table. Each representative ASV was assigned to different taxa levels against the SILVA 132 release (Quast et al., 2013). Multiple sequence alignments were performed using micca_msa, followed by a rooted phylogenetic tree construction in micca_rooted_tree (v1.7.0) (Albanese et al., 2015). Each sample of gut, water, and detritus was set to a uniform lowest depth of 7495 bp for the calculation of alpha-beta diversity and microbial community composition.

#### Downstream Bioinformatics

Alpha diversity regarding richness, Fisher-alpha, and Shannon and Simpson indices were calculated in microbiomeSeq.^1^ nMDS was used to display beta-ordination in terms of Bray–Curtis dissimilarity of relative abundance in phyloseq R package (McMurdie and Holmes, 2013), while permutational multivariate analysis of variance (PERMANOVA) was performed using the vegan R package (Dixon, 2003). Relative abundances of bacteria at phylum and genus levels were calculated in phyloseq and ampvis2 (Andersen et al., 2018) R packages. Metagenome prediction from the 16S rRNA ASV dataset was performed using the Picrust2 algorithm in support of KEGG pathway descriptions (Douglas et al., 2020).

### Gene Expression Analysis

Based on recent studies on gene expression analysis (Gupta et al., 2020; Siddik et al., 2020; Chaklader et al., 2021b) of *L. calcarifer* after feeding trials and immune-related transcriptome analysis, one anti-inflammatory and four pro-inflammatory cytokines including tumor necrosis factor-alpha (TNF-α) and interleukins IL-1, IL-8, IL-10, and IL-17 were tested for their relative expression in real-time PCR. We have selected cytokines for this study for expression analysis as they are the mediators of immune response and indicators of stress and infection (Reyes-Cerpa et al., 2012). For the gene expression, gut samples (*n* = 4) from each group were preserved in RNAlater according to the manufacturer’s instructions and stored at –80°C until processing. The gut tissue after removing the intestinal content was used for RNA extraction, and the same fish was used for both DNA and RNA extraction. The samples were thawed on ice, followed by RNA extraction using an RNeasy Plus Mini Kit (Qiagen, Hilden, Germany) by following the manufacturer’s instructions for tissue samples. Digestion of DNA and removal of enzymes were performed using a TURBO DNA-free™ Kit (Thermo Fisher Scientific, United States). An RNeasy MiniElute Cleanup Kit (Qiagen, Hilden, Germany) was used for the purification of RNA. Quality of the extracted RNA was checked using 1% agarose gel. The RNA concentration was measured in a Qubit 4 Fluorometer (Thermo Fisher Scientific, United States) and a NanoDrop 2000c Spectrophotometer (Thermo Fisher Scientific, Waltham, MA, United States). The presence of any DNA inhibitors was checked further with PCR amplification of bacterial universal 16S, 27F, and 1492R. The first strand cDNA was synthesized using a SuperScript™ IV First-Strand Synthesis System (Thermo Fisher Scientific, United States). Quantitative real-time PCR was performed using PowerUp™ SYBR Master Mix (Thermo Scientific, United States) and gene primers with a CFX96 Real-Time PCR Detection System (Bio-Rad Laboratories Inc., United States). The relative expression level of each gene was calculated using the 2^–ΔΔCT^ method, followed by normalization against the β-actin reference gene (Livak and Schmittgen, 2001).

### Statistical Analysis

One-way ANOVA, followed by Tukey’s HSD, was used to compare alpha diversity among the groups. Non-parametric statistical analysis of the distance metric was performed using ANODIS with 1000 permutations. Differential abundance of microbial communities at the genus level was calculated using the Kruskal–Wallis test, followed by a Dunn *post hoc* with Bonferroni adjustment.

Significantly altered metabolic pathways were identified by linear discriminant analysis (LDA), with a stringent LDA cut-off value of ≥4.0 used to compare the functional features of microbial compositions. At all stages, a *p*-value of <0.05 was considered statistically significant. The “Pearson” correlation coefficient of taxa abundance and dietary variables were calculated using the microbiomeSeq R package.

## Results and Discussion

### Characterization of Oils

The two oils we have chosen for this study are chemically very different. HFO is highly sulfurous (102 mg/kg) compared to ACO (3.9 mg/kg) (Table 1 and Supplementary Table 2). The PAH profiles of the two oils are also dissimilar: ACO has higher levels of bicyclic aromatics (491 mg/kg) than HFO (245 mg/kg), similar levels of tricyclic aromatics (160 and 150 mg/kg, respectively), and lower levels of the higher molecular weight tetracyclic (4.5 and 29 mg/kg, respectively) and pentacyclic aromatic compounds (0.87 and 19 mg/kg, respectively). In all crude oils, the concentration of metals varies greatly (Yasnygina et al., 2006; Pereira et al., 2010). Metals of note present in HFO are iron (37.9 mg/kg), vanadium (15.7 mg/kg), nickel (12.2 mg/kg), cobalt (2.15 mg/kg), and zinc (1.19 mg/kg). ACO contains less iron (4.73 mg/kg), nickel (0.11 mg/kg), and no vanadium or cobalt. ACO contains slightly lower amounts of aluminum (10.2 mg/kg) than HFO (15.4 mg/kg) and similar low levels of tin (0.18 and 0.13 mg/kg, respectively). The dosage of PAHs and metals in the fish feeds used in the study is summarized in Table 1.

### Sequence Statistics

A total of 5.6 million quality reads were obtained from 86 samples of fish feed, tank water and gut samples. For gut samples alone, 4.2 million quality reads were obtained. The reads generated 6104 ASVs, which were classified into 14 phyla and 478 genera. In the gut, 6104 ASVs were classified into 14 phyla and 336 genera. The average number of reads (52,486.2 ± 14,592.8), Good’s coverage index (0.997 ± 0.001), and rarefaction curve (Supplementary Figure 1) indicated that each sample was sequenced at a high depth to capture maximum bacteria at various taxa levels.

### Alpha-Beta Diversity of Water, Feed, and Fish Gut Microbiota

**FIGURE 1:**
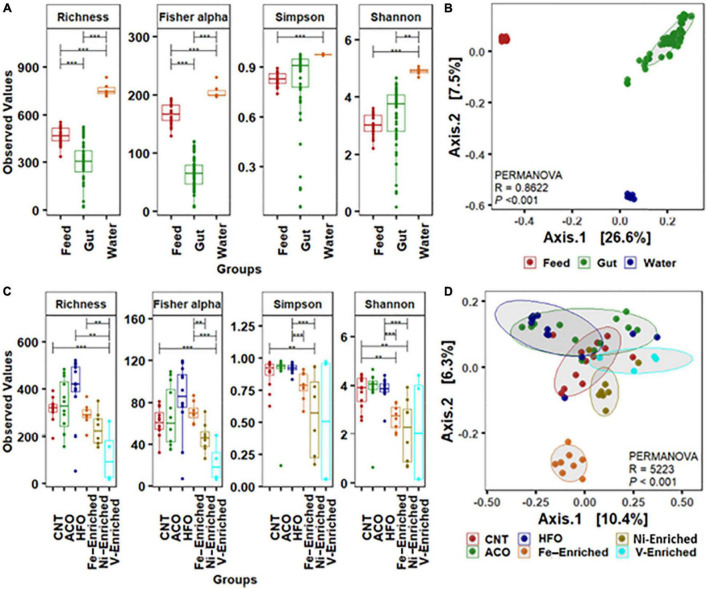
Alpha-beta diversity of bacterial community. **(A)** Alpha diversity measurements of bacterial communities in the gut, water, and feed. **(B)** Beta ordination showing clustering of bacterial amplicon sequence variants (ASVs) in the gut, water, and feed. **(C)** Alpha-diversity measurements of bacterial communities in the gut with six different dietary treatments. **(D)** Beta ordination showing clustering of bacterial ASVs in the gut of barramundi fed six different diets. Abbreviations: Neg, negative control; ACO, Australian crude oil; HFO, heavy fuel oil. *Significant at an α-level of 0.05. **Significant at an α-level of 0.005. ***Significant at an α-level of 0.001.

The water and feed had significantly higher bacterial diversity (Figure 1A) and composed of completely different bacterial groups compared to the gut microbiome, as revealed by beta-ordination (Figure 1B). Rearing water harbors diverse and different groups of bacteria (Qin et al., 2016), suggesting little or no correlation to the fish gut bacterial communities (Giatsis et al., 2015; Zeng et al., 2020). The gut microbial signatures in the juvenile stage are considered a valuable indicator of fish health and immunity (Talwar et al., 2018), and the composition of gut bacteria is highly influenced by dietary intervention (Parata et al., 2020; Nguyen et al., 2021; Serra et al., 2021). Since the water quality and other experimental parameters like temperature, pH, salinity, and dissolved oxygen remained constant throughout the trial, the shift of microbial communities in the gut of fish in this study primarily arises from the PAH- and metal-enriched diets used to feed juvenile *L. calcarifer*.

### Alpha-Beta Diversity of Fish Gut Microbiota Following Petroleum Hydrocarbon and Metal Exposure

Based on the weighted (relative abundance) UniFrac metric, analysis of gut bacteria from the six different test groups revealed significant reduction of the bacterial diversity in the gut of fish exposed to V, Ni, and Fe, whereas no differences were observed in the groups fed crude oil compared to negative control fish fed the unaltered diet (Figure 1C). The alpha diversity including microbial richness or species diversity and Fisher-alpha in the gut of fish fed the V-enriched diet was significantly lower compared to that of the negative control group (*p* < 0.001). The Fisher-alpha diversity index of V-enriched diet fish was also significantly less than that of negative controls. The Shannon diversity index, generally a better predictor when the sample size is a large proportion of the whole population (Beck and Schwanghart, 2010), showed that the microbiome of fish fed the Ni- and Fe-enriched diets was also significantly less diverse that of the negative control group (*p* < 0.005). Centroid analysis of beta-dispersion showed distinctly different bacteria in Fe-enriched diet groups in both weighted and unweighted UniFrac metrics compared to other groups in the present study (Figure 1D).

Legrand et al. (2020) showed that the fish microbiome diversity decreases with the progression of gut enteritis. The decrease in diversity and dissimilar microbes in the present study (Figure 1C and Supplementary Table 3) may indicate reductions in the overall gut health of fish challenged with metal-enriched diets. Further research on histological changes exhibited by the intestinal tissues would be required to confirm this.

### Microbial Composition in the Fish Gut Following Petroleum Hydrocarbon and Metal Exposure

#### At the Phylum Level

The negative control test group showed that the normal microbiome of *L. calcarifer* on a commercial fishmeal diet contains core taxa of phyla Proteobacteria (62%), Firmicutes (7%), Planctomycetes (3%), Actinobacteria (2%), and Bacteroidetes (1%). This is typical and agrees with other microbiome studies on *L. calcarifer* (Gupta et al., 2019; Zheng et al., 2019; Chaklader et al., 2021a) and other species (Ghanbari et al., 2015; Adamovsky et al., 2018; DeBofsky et al., 2020).

All metal-enriched feeds produced highly significant changes in the fish microbiome through the alteration of bacterial richness for Proteobacteria, Firmicutes, and Bacteroidetes. These three bacterial phyla are mainly associated with the metabolism, nutrient assimilation, and immunity of host fish. Previous reports have shown that fish exposed to metals demonstrate significantly altered bacterial abundance with complete disruption of Proteobacteria and Bacteroidetes following long-term exposure (Meng et al., 2018; Kakade et al., 2020). Fish fed a Ni-enriched diet had a microbiome dominated by Proteobacteria (81.1%), with no other individual phyla comprising more than 2% of the microbiome. In addition to fish, this effect of dietary Ni altering the relative abundance of Firmicutes and Bacteroidetes has also been shown in rats (Richardson et al., 2018).

Firmicutes comprise 67.9% of bacteria in the microbiome of V-exposed fish. V is generally the most toxic metal included in this study, and Firmicutes is the most resistant taxa in the fish gut that can survive in extreme environments with higher concentration of metals (Xia et al., 2018; Kakade et al., 2020). This increase in the abundance of Firmicutes bacteria may be due to vanadium resistance. Firmicutes are mainly carbohydrate-metabolizing and butyrate-producing bacteria linked to the nutrition and energy of epithelial and gastrointestinal cells, assisting in reducing the carcinogenic and inflammatory effect of metals (Collinder et al., 2003; Kakade et al., 2020). Higher Firmicutes abundance suggests restoration of the intestinal barrier function by the fish to maintain its health and immune performance.

**FIGURE 2:**
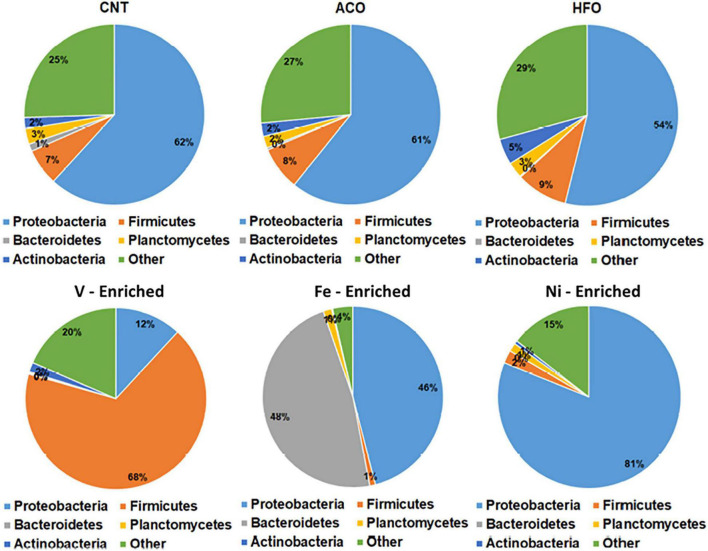
Relative abundance of bacteria at the phylum level in the gut of barramundi with six different diets. Abbreviations: ACO, Australian crude oil; HFO, heavy fuel oil.

Bacteroidetes and Proteobacteria comprised 47.7 and 46% of total ASVs respectively in the Fe-enriched group, whereas no other group had more than 1% read abundance (Figure 2). This exposure shows complete dysbiosis of Firmicutes, a phylum that links metabolism and immunity in aquatic species (Foysal et al., 2020; Gaudioso et al., 2021). Bacteroidetes are involved in nutrient absorption and epithelial cell maturation of fish (Evariste et al., 2019). Other reports indicate that exposure to cadmium results in a similar dominance by Bacteroidetes and Proteobacteria in the microbiome of Nile tilapia (*Oreochromis niloticus*) (Zhai et al., 2017; Meng et al., 2018).

Unlike the metal-enriched diets, petroleum hydrocarbons had no pronounced effects on gut phyla with similar relative abundances of Proteobacteria, Firmicutes, and Bacteroidetes in the ACO, and HFO test groups compared to negative controls.

#### At the Genus Level

**FIGURE 3:**
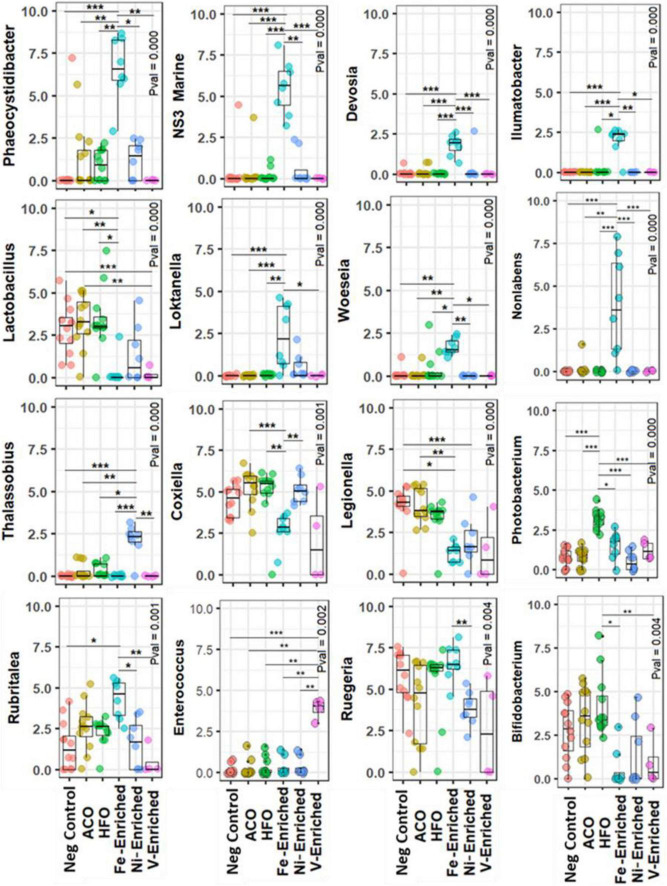
Differential abundance of bacteria at the genus level in the gut of juvenile barramundi exposed to petroleum hydrocarbon and heavy metals. Rarefied abundances were log1p-transformed for generating plots Calculated using the Kruskal–Wallis along with the post hoc Dunn test. The P-value was adjusted with Bonferroni correction. *Significant at an α-level of 0.05. **Significant at an α-level of 0.005. ***Significant at an α-level of 0.001. Abbreviations: ACO, Australian crude oil; HFO, heavy fuel oil.

**FIGURE 4:**
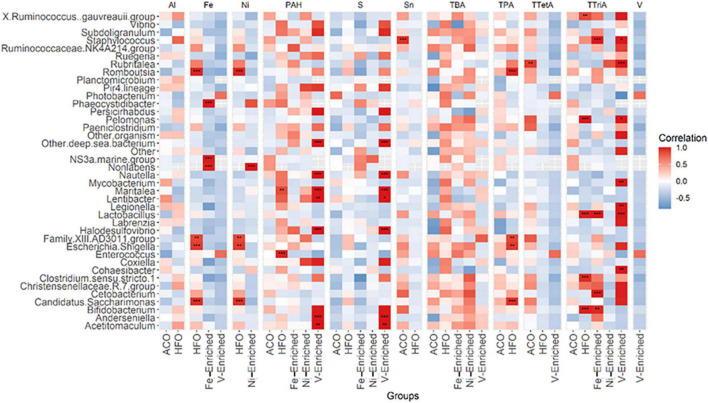
Pearson correlation between 40 abundant genera in fish microbiome and five categories of petroleum hydrocarbons and three metals in diets. The color code at the right indicates type and degree of correlation. *Significant at an α-level of 0.05. **Significant at an α-level of 0.005. ***Significant at an α-level of 0.001. Abbreviations: CNT, control; ACO, Australian crude oil; HFO, heavy fuel oil; Al, aluminum; Fe, iron; Ni, nickel; PAH, total aromatic hydrocarbon; S, sulfur; Sn, tin; TBA, total bicyclic aromatics; TPA, total pentacyclic aromatics; TTetA, total tetracyclic aromatics; TTriA, total tricyclic aromatics; V, vanadium.

In the gut microbiome of the negative control group, *Ruegeria* and *Escherichia-Shigella* were the most abundant bacteria genera, whereas both crude oil test groups favored the growth of *Photobacterium* and, to a lesser extent, *Bifidobacterium* (Figure 3 and Supplementary Figure 3). *Photobacterium, Ruegeria*, and *Escherichia-Shigella* had a read abundance of >1% in all samples, regardless of treatments and conditions; therefore, these three genera can be defined as “core” or “resident” bacteria in juvenile *L. calcarifer*. *Photobacterium* abundance increased in all treatment groups, compared to control (Figure 4). Among metal-exposed fish, *Phaeocystidibacter* was enriched exclusively in the Fe-enriched group, and *Enterococcus* was enriched solely in the V-enriched group (although there was variation in the distribution of reads for the genus *Enterococcus* within the V-enriched group). Also, in the gut of fish fed the Fe-enriched diet, *Phaeocystidibacter, NS3 marine, Devosia, Ilumatobacter, Loktanella*, and *Woeseia* had a significantly higher abundance than the microbiomes of negative control and crude oil-exposed fish. A Ni-enriched diet increased the abundance of *Coxiella, Escherichia-Shigella, Thalassobius*, and *Cohaesibacter*.

Compared to negative control fish, *Lactobacillus* and *Legionella* were significantly reduced in the microbiomes of fish exposed to Ni, V, and Fe, but not to petroleum hydrocarbons (Figure 3 and Supplementary Figure 2).

A recent study by Hano et al. (2021) showed that *Photobacterium* is enriched in the gut of red sea bream (*Pagrus major*) following exposure to phenanthrene (a tricyclic PAH) and proposed microbiome analysis as a possible biomarker for phenanthrene exposure. However, our results indicate that an increase in the relative abundance of *Photobacterium* is likely not specific to phenanthrene, but also other higher molecular weight PAHs such as pyrenes and benzo(*a*)pyrenes as well, given the higher concentrations of these 4- and 5-ring compounds in HFO than in ACO. Another study reported high percentages of Vibrionales, mainly *Vibrio* and *Photobacterium*, in red snapper (*Lutjanus campechanus*) following DWH oil spill in Louisiana, composed of 32 and 23% of the total genera (Arias et al., 2013). A very similar shift in the relative abundance was observed in our study, despite a higher cumulative abundance of *Photobacterium* (19.6%) in the ACO and HFO groups than *Vibrio* (6.2%). With only 1.6 and 0.3% of *Photobacterium* and *Vibrio* abundance in control, the shift of these two genera in the ACO and HFO groups was therefore very distinct. Based on the observation, we can presume that *Photobacterium* increased in relative abundance in a dose-dependent manner relative to the combined total tri-, tetra-, and pentacyclic PAHs.

Some genera known to be able to metabolize PAHs as their only energy source such as *Vibrio* (Walter et al., 2019) were enriched in fish fed petroleum hydrocarbon-enriched diets, but curiously other oil-metabolizing bacteria such as *Mycobacterium* (Walter et al., 2019) were not (Supplementary Figure 2). Other genera capable of PAH degradation, such as *Sphingomonas* (Pinyakong et al., 2003; Milan et al., 2018; Walter et al., 2019), reported in the microbiome of wild fish populations exposed to petroleum hydrocarbons (Walter et al., 2019) were not detected in our study probably because these genera are never introduced to the microbiome of nursery-raised fish.

*Phaeocystidibacter* has been found to be enriched in microbial communities exposed to fluorene and benzo(*a*)pyrene (Kahla et al., 2021). These compounds are present in the HFO, Ni-enriched, and V-enriched test groups, none of which exhibited increases in the relative abundance of *Phaeocystidibacter*. Conversely, this genus was notably increased in the Fe-enriched test group, which was the only group in the present study to contain neither of these large molecular weight PAHs. This highlights the challenge presented by the inherent variability of microbiome analysis at the genus level.

### Metagenome Predictions

**FIGURE 5:**
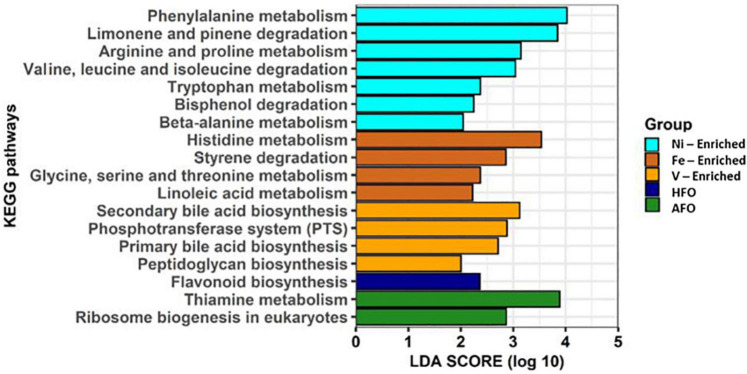
Predicted functional features of 16S rRNA metagenomic data using Picrust2. Abbreviations: ACO, Australian crude oil; HFO, heavy fuel oil.

Alterations in predicted metabolic pathways were observed among the different treatment groups. Most of the significant changes found in functional features were linked to exposure to metals. A diet containing ACO and HFO was linked to only three of 18 significantly enriched metabolic pathways. While fish exposed to Fe- and Ni-enriched diet responded mostly with perturbations in the metabolism and degradation of amino acids, fish fed a V-enriched diet showed metabolic changes linked to the biosynthesis of bile acid and peptidoglycan. Other upregulated metabolic pathways are flavonoid biosynthesis in HFO, and thiamine metabolism and ribosome biogenesis in the ACO group (Figure 5).

Although Ni is a necessary co-factor for many enzymes (Boer et al., 2014; Alfano and Cavazza, 2020), it appears to have no part in any of the amino acid metabolism which would explain the functional changes predicted in the microbiome of the Ni-enriched test group. Likewise, Fe and V have numerous roles in cellular biochemistry (reviewed by Beard et al., 1996; Gustafsson, 2019), but how they might influence, for example, amino acid metabolism, the breakdown of styrenes or the formation of the peptidoglycan sheath on the bacterial cell wall is not mentioned in current literature. While these links are reported for the first time, a causal relationship could not at this point be established between dietary exposure to petroleum hydrocarbons or to metals.

### Taxa-Environmental Correlations

A total of 27 genera in the gut were found to be influenced by various petroleum hydrocarbons and metals in the diet (Figure 4). Of these, only 11 genera, namely, *Staphylococcus, Rubritalea, Phaeocystidibacter*, NS3 marine bacteria, *Non-labens, Nautella, Lactobacillus, Escherichia-Shigella, Enterococcus, Cetobacterium*, and *Bifidobacterium*, had more than 1% of read abundance in at least one of the groups in the trial. The correlation plot shows *Phaeocystidibacter, NS3 marine*, and *Non-labens* preferred a higher concentration of Fe for their growth and multiplication, whereas an inverse association was observed between *Lactobacillus* and Fe concentration (Figure 4). A positive association was also identified between *Thalassobius* and Ni concentration.

### Gene Expression

**FIGURE 6:**
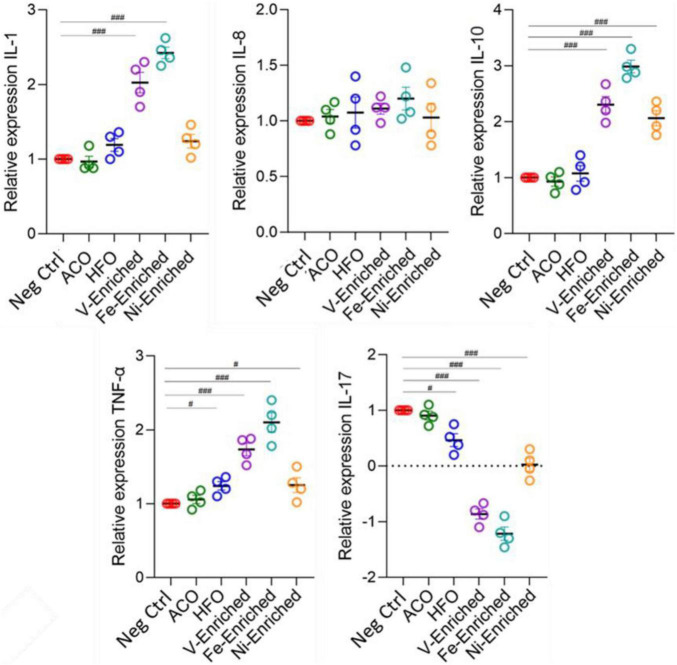
Expression of cytokine genes (fold changes relative to negative control) in the gut of juvenile *Lates calcarifer* exposed to petroleum hydrocarbons and metals. One-way ANOVA with the Dunnett *post hoc* test was carried out. ^#^Significant at an α-level of 0.05. ^##^Significant at an α-level of 0.005. ^###^Significant at an α-level of 0.001. Abbreviations: ACO, Australian crude oil; HFO, heavy fuel oil.

Cytokines are important markers to analyze fish health and immunity. The gene expression data showed upregulation of pro-inflammatory cytokines IL-1, IL-10, and TNF-α in fish fed V- and Fe-enriched diets. Upregulation of IL-10 and TNF-α was also seen in the Ni-enriched test group, and TNF-α alone was upregulated in fish exposed to dietary HFO. Downregulation of the anti-inflammatory IL-17 cytokine relative to negative control fish was observed in all test groups, except in the ACO-fed treatment group. Compared to the negative control group, no changes in the relative expression level of IL-8 were observed in any of the test groups (Figure 6).

In aquaculture, the use of probiotic dietary supplements is intended to improve fish health. Changes in cytokine expression in response to probiotic supplements in diets (mainly *Lactobacillus*) has been reported in zebrafish (*Danio rerio*) (He et al., 2017; Perry et al., 2020), carp (*Cyprinus carpio*) (Giri et al., 2018), rainbow trout (*Oncorhynchus mykiss*) (Nikoskelainen et al., 2003), and crayfish marron (*Cherax cainii*) (Foysal et al., 2020). However, the Ni- and Fe-enriched dietary groups that showed the largest changes in cytokine expression also evidenced an associated significant reduction of *Lactobacillus* abundance (*p* < 0.05) in response to dietary metals exposure (Supplementary Figure 2). Such a trend was also present (non-significantly) in the V-enriched dietary group. This pattern of elevated expression of pro-inflammatory cytokines and an associated reduction in the relative abundance of gut microbiome *Lactobacillus* has been reported in carp exposed to trichlorfon, an organophosphorus pesticide used for parasite control in aquaculture (Chang et al., 2020).

Part of the normal microbiome of healthy fish (Ringø and Gatesoupe, 1998; Balcázar et al., 2007; Gómez and Balcázar, 2008), *Lactobacillus* has been shown to reduce the pathogenic effects of lead (Giri et al., 2018) and cadmium (Zhai et al., 2017) and inhibit pathogenic bacterial species (He et al., 2017; Collins, 2019). It may be that the very low abundance of *Lactobacillus* in the gut microbiota of fish may be useful as a biomarker of exposure to some specific toxicants such as metals and some pesticides, but not petroleum hydrocarbons. Conversely, another widely studied lactic acid bacterium, *Bifidobacterium*, positively correlated with petroleum hydrocarbon exposure (Figure 3) and may also be a biomarker candidate worthy of future study alongside *Photobacterium*.

## Conclusion

We have demonstrated that the gut microbiome of fish exposed *via* diet to crude oils and V, Ni, and Fe undergoes significant changes. In general, dietary metal exposure produced a greater reduction in diversity and elevated immune response than petroleum hydrocarbons.

Analysis of the microbiome at the phylum level provides clear indications of exposure to V, Ni, and Fe, and to a lesser extent at the genus level where the picture is more complicated. The phylum Firmicutes is greatly emphasized in the microbiome of fish exposed to vanadium, and Protobacteria is enriched in response to Ni. Exposure to Fe increases the abundance of Bacteroidetes, but decreases Protobacteria. At the genus level, enhanced *Photobacterium* in the fish microbiome shows potential as a biomarker of exposure to PAHs, increasing proportionately to the dietary concentration of higher molecular weight PAHs. Reductions in *Lactobacillus* may also be a candidate as a biomarker for exposure to metals, and possibly other toxicants. However, the percentage of uncultured, unclassified, and ambiguous taxa that have been classified as “other” was one of the major limitations of this study. Nevertheless, the size of the samples used for data analysis from the fish gut and different sources was sufficient enough to make a conclusive statement on gut microbial signatures following petroleum hydrocarbon and metal exposures. Future studies are needed to further explore the potential of gut microbiome analysis as a biomarker for petroleum hydrocarbons, metals, other toxicants, or as a general indicator of fish health.

## Data Availability Statement

The datasets presented in this study can be found in online repositories. The names of the repository/repositories and accession number(s) can be found below: https://www.ncbi.nlm.nih.gov/, PRJNA785175.

## Ethics Statement

The animal study was reviewed and approved by Curtin University Animal Ethics Committee (Approval Number: ARE2019-11).

## Author Contributions

FS: conceptualization, investigation, methodology, visualization, roles and writing–original draft, and writing–review and editing. MF: data curation, formal analysis, investigation, methodology, software, validation, roles and writing–original draft, and writing–review and editing. AT: methodology, resources, supervision, and writing–review and editing. MG: conceptualization, funding acquisition, methodology, project administration, resources, supervision, and writing–review and editing. All authors contributed to the article and approved the submitted version.

## Conflict of Interest

The authors declare that the research was conducted in the absence of any commercial or financial relationships that could be construed as a potential conflict of interest.

## Publisher’s Note

All claims expressed in this article are solely those of the authors and do not necessarily represent those of their affiliated organizations, or those of the publisher, the editors and the reviewers. Any product that may be evaluated in this article, or claim that may be made by its manufacturer, is not guaranteed or endorsed by the publisher.

## Footnotes

1
https://github.com/umerijaz/microbiomeSeq
