# Supplementary material for: Gut Microbiome as a Potential Biomarker in Fish: Dietary Exposure to Petroleum Hydrocarbons and Metals, Metabolic Functions and Cytokine Expression in Juvenile Lates calcarifer
Source: Front Microbiol. 2022 Jul 22;13:827371. doi: 10.3389/fmicb.2022.827371 (PMC9356228; doi:10.3389/fmicb.2022.827371)
Supplement: Supplementary file 1 [file Data_Sheet_1.PDF]

SUPPLEMENTARY INFORMATION

Supplementary Materials:

Table S1: Mixtures of Metal-enriched feeds plus a selection of petroleum hydrocarbons

	Compound	Concentration in Fish Feed (mg/kg)
**Mix A**	**Vanadium (as V_2O_5)**	**20**
	Naphthalene	1.17
	Phenanthrene	0.63
	Diphenylmethane	6.75
	Dibenzothiophene	0.63
	Fluorene	2.25
	Pyrene	1.80
	Biphenyl	3.69
	Decalin	2.43
	Adamantane	2.61
	Tridecane	4.50
**Mix B**	**Iron (as $FeSO_4$)**	**500**
	1-Methylnaphthalene	1.26
	3,6-Dimethylphenanthrene	0.18
	1-Pheny-dodecane	4.50
	Iso-butyl-benzene	3.09
	Retene	0.33
	1,3-Di-isopropylbenzene	3.15
	2-Methyl-indene	4.05
	Phytane	4.50
	Pristane	0.90
	Heptadecane	4.50
	Octadecane	4.50
**Mix C**	**Nickel (as Ni_2SO_4)**	**500**
	1, 5-Dimethynaphthalene	2.97
	Benzo(a)pyrene	1.08
	1-Methyl-fluorene	1.26
	2-Iso-propyl-naphthalene	1.53
	Indane	4.50
	Tetralin	5.13
	1,3-Dimethyladamantane	4.41
	2-methylbiphenyl	2.25

Table S2: Metals Analysis of an Australian Crude Oil (ACO) and Heavy Fuel Oil (HFO).

Element	ACO (1) (mg/kg)	ACO (2) (mg/kg)	ACO (3) (mg/kg)	ACO Mean (mg/kg)	Std Err	HFO (1) (mg/kg)	HFO (2) (mg/kg)	HFO (3) (mg/kg)	HFO Mean (mg/kg)	Std Err
Aluminium	30.70	< 0.5	< 0.5	10.23	-	8.15	33.30	4.86	15.44	8.98
Antinomy	< 0.01	< 0.01	< 0.01	< 0.01	-	0.43	0.45	0.50	0.46	0.02
Arsenic	< 0.03	< 0.03	< 0.03	< 0.03	-	0.03	0.03	0.06	0.04	0.01
Barium	0.25	0.05	0.03	0.11	0.06	1.18	1.32	1.45	1.32	0.08
Beryllium	< 0.01	< 0.01	< 0.01	< 0.01	-	< 0.01	< 0.01	< 0.01	< 0.01	-
Bismuth	< 0.01	< 0.01	< 0.01	< 0.01	-	< 0.01	< 0.01	< 0.01	< 0.01	-
Boron	< 1.7	< 1.7	< 1.7	< 1.7	-	< 1.7	< 1.7	< 1.7	< 1.7	-
Cadmium	< 0.01	< 0.01	0.01	0.01	-	< 0.01	< 0.01	< 0.01	< 0.01	-
Caesium	< 0.01	< 0.01	< 0.01	< 0.01	-	< 0.01	< 0.01	< 0.01	< 0.01	-
Calcium	91.00	11.70	10.50	37.73	26.64	11.20	72.70	3.10	29.00	21.9
Cerium	< 0.01	< 0.01	< 0.01	< 0.01	-	0.00	0.02	0.00	0.01	0.01
Chromium	0.89	< 0.12	< 0.12	0.89	-	0.45	0.28	0.00	0.24	0.13
Cobalt	< 0.46	< 0.46	< 0.46	< 0.46	-	< 0.46	0.66	3.63	2.15	1.48
Copper	0.45	< 0.31	< 0.31	0.45	-	< 0.31	< 0.31	< 0.31	< 0.31	-
Dysprosium	< 0.01	< 0.01	< 0.01	< 0.01	-	< 0.01	< 0.01	< 0.01	< 0.01	-
Erbium	< 0.01	< 0.01	< 0.01	< 0.01	-	< 0.01	< 0.01	< 0.01	< 0.01	-
Europium	< 0.01	< 0.01	< 0.01	< 0.01	-	< 0.01	< 0.01	< 0.01	< 0.01	-
Gadolinium	< 0.01	< 0.01	< 0.01	< 0.01	-	< 0.01	< 0.01	< 0.01	< 0.01	-
Gallium	< 0.01	< 0.01	< 0.01	< 0.01	-	< 0.01	0.02	0.02	0.02	0.00
Germanium	< 0.01	< 0.01	< 0.01	< 0.01	-	< 0.01	< 0.01	< 0.01	< 0.01	-
Hafnium	< 0.01	< 0.01	< 0.01	< 0.01	-	< 0.01	< 0.01	< 0.01	< 0.01	-
Holmium	< 0.01	< 0.01	< 0.01	< 0.01	-	< 0.01	< 0.01	< 0.01	< 0.01	-
Indium	< 0.01	< 0.01	< 0.01	< 0.01	-	< 0.01	< 0.01	< 0.01	< 0.01	-
Iron	7.65	1.29	5.25	4.73	1.85	37.30	40.70	35.70	37.90	1.47
Lanthanum	< 0.01	< 0.01	< 0.01	< 0.01	-	0.71	0.69	0.89	0.77	0.06
Lead	0.08	0.09	0.09	0.08	0.00	0.02	0.08	0.04	0.04	0.02
Lithium	< 0.05	< 0.05	< 0.05	< 0.05	-	< 0.05	0.11	< 0.05	0.11	-
Lutetium	< 0.01	< 0.01	< 0.01	< 0.01	-	< 0.01	< 0.01	< 0.01	< 0.01	-
Magnesium	3.06	0.53	< 0.15	1.80	1.04	1.43	2.73	1.24	1.80	0.47
Manganese	< 0.04	< 0.04	< 0.04	< 0.04	-	< 0.04	< 0.04	< 0.04	< 0.04	-
Mercury	< 0.01	< 0.01	< 0.01	< 0.01	-	< 0.01	< 0.01	< 0.01	< 0.01	-
Molybdenum	< 0.01	< 0.01	< 0.01	< 0.01	-	0.05	0.06	0.06	0.05	0.00
Neodymium	< 0.01	< 0.01	< 0.01	< 0.01	-	< 0.01	< 0.01	< 0.01	< 0.01	-
Nickel	0.14	< 0.06	0.08	0.11	0.06	10.90	12.50	13.30	12.23	0.71
Niobium	< 0.03	< 0.03	< 0.03	< 0.03	-	< 0.03	< 0.03	< 0.03	< 0.03	-
Phosphorous	< 3.5	< 3.5	< 3.5	< 3.5	-	< 3.5	< 3.5	< 3.5	< 3.5	-
Potassium	38.90	35.40	36.00	36.77	1.08	4.50	12.50	7.37	8.12	2.34
Praseodymium	< 0.01	< 0.01	< 0.01	< 0.01	-	< 0.01	< 0.01	< 0.01	< 0.01	-
Rubidium	0.14	0.02	< 0.01	< 0.01	0.27	0.07	0.16	0.07	0.10	0.03
Samarium	< 0.01	< 0.01	< 0.01	< 0.01	-	< 0.01	< 0.01	< 0.01	< 0.01	-

Element	ACO (1) (mg/kg)	ACO (2) (mg/kg)	ACO (3) (mg/kg)	ACO Mean (mg/kg)	Std Err	HFO (1) (mg/kg)	HFO (2) (mg/kg)	HFO (3) (mg/kg)	HFO Mean (mg/kg)	Std Err
Selenium	0.13	< 0.01	0.06	0.09	0.03	0.00	0.02	0.00	0.01	0.01
Silicon	< 1	< 1	< 1	< 1	-	< 1	< 1	< 1	< 1	-
Silver	< 0.01	< 0.01	< 0.01	< 0.01	-	< 0.01	< 0.01	< 0.01	< 0.01	-
Sodium	4.63	< 2.5	< 2.5	4.63	-	< 2.5	< 2.5	< 2.5	< 2.5	-
Strontium	0.61	0.07	< 0.01	0.34	0.27	0.26	0.74	0.30	0.43	0.15
Sulfur	335	460	386	394	36.2	11900	9800	9060	10253	851
Tantalum	< 0.01	< 0.01	< 0.01	< 0.04	-	< 0.01	< 0.01	< 0.01	< 0.01	-
Terbium	< 0.01	< 0.01	< 0.01	< 0.01	-	< 0.01	< 0.01	< 0.01	< 0.01	-
Thallium	< 0.01	< 0.01	< 0.01	< 0.01	-	< 0.01	< 0.01	< 0.01	< 0.01	-
Thorium	< 0.01	< 0.01	< 0.01	< 0.01	-	< 0.01	< 0.01	< 0.01	< 0.01	-
Thulium	< 0.01	< 0.01	< 0.01	< 0.01	-	< 0.01	< 0.01	< 0.01	< 0.01	-
Tin	0.03	< 0.01	0.32	0.18	0.10	0.19	0.02	0.18	0.13	0.06
Titanium	< 0.24	< 0.24	< 0.24	< 0.24	-	3.03	3.47	3.22	3.24	0.13
Tungsten	< 0.04	< 0.04	< 0.04	< 0.01	-	< 0.04	< 0.04	< 0.04	< 0.04	-
Uranium	< 0.01	< 0.01	< 0.01	< 0.01	-	< 0.01	< 0.01	< 0.01	< 0.01	-
Vanadium	< 0.03	< 0.03	< 0.03	< 0.03	-	14.20	14.60	17.00	15.27	0.87
Ytterbium	< 0.01	< 0.01	< 0.01	< 0.01	-	< 0.01	< 0.01	< 0.01	< 0.01	-
Yttrium	< 0.01	< 0.01	< 0.01	< 0.01	-	< 0.01	< 0.01	< 0.01	< 0.01	-
Zinc	1.58	1.60	1.24	1.47	0.85	0.97	1.41	1.20	1.19	0.13
Zirconium	0.22	< 0.01	< 0.01	0.22	0.13	< 0.01	< 0.01	< 0.01	< 0.01	-

Table S3. Beta-ordination PERMANOVA (P_{anodis}) for Fe- and Ni-enriched groups

Ordination (Weighted)	P-value	Ordination (Unweighted)	P-value
Fe-enriched vs ACO	4.86E-08	Fe-enriched vs ACO	5.48E-04
Fe-enriched vs HFO	2.36E-06	Fe-enriched vs HFO	2.36E-04
Fe-enriched vs CNT	6.72E-06	Fe-enriched vs CNT	8.67E-05
Fe-enriched vs V-enriched	3.22E-05	Fe-enriched vs V-enriched	1.92E-03
Fe-enriched vs Ni-enriched	6.88E-04	Fe-enriched vs Ni-enriched	5.44E-02
Ni-enriched vs ACO	2.32E-05	Ni-enriched vs ACO	5.08E-04
Ni-enriched vs HFO	4.56E-05	Ni-enriched vs HFO	1.06E-03
Ni-enriched vs Fe-enriched	6.36E-04	Ni-enriched vs Fe-enriched	4.34E-04
Ni-enriched vs CNT	1.88E-03	Ni-enriched vs CNT	1.96E-04
Ni-enriched vs V-enriched	2.12E-03	Ni-enriched vs V-enriched	1.02E-02

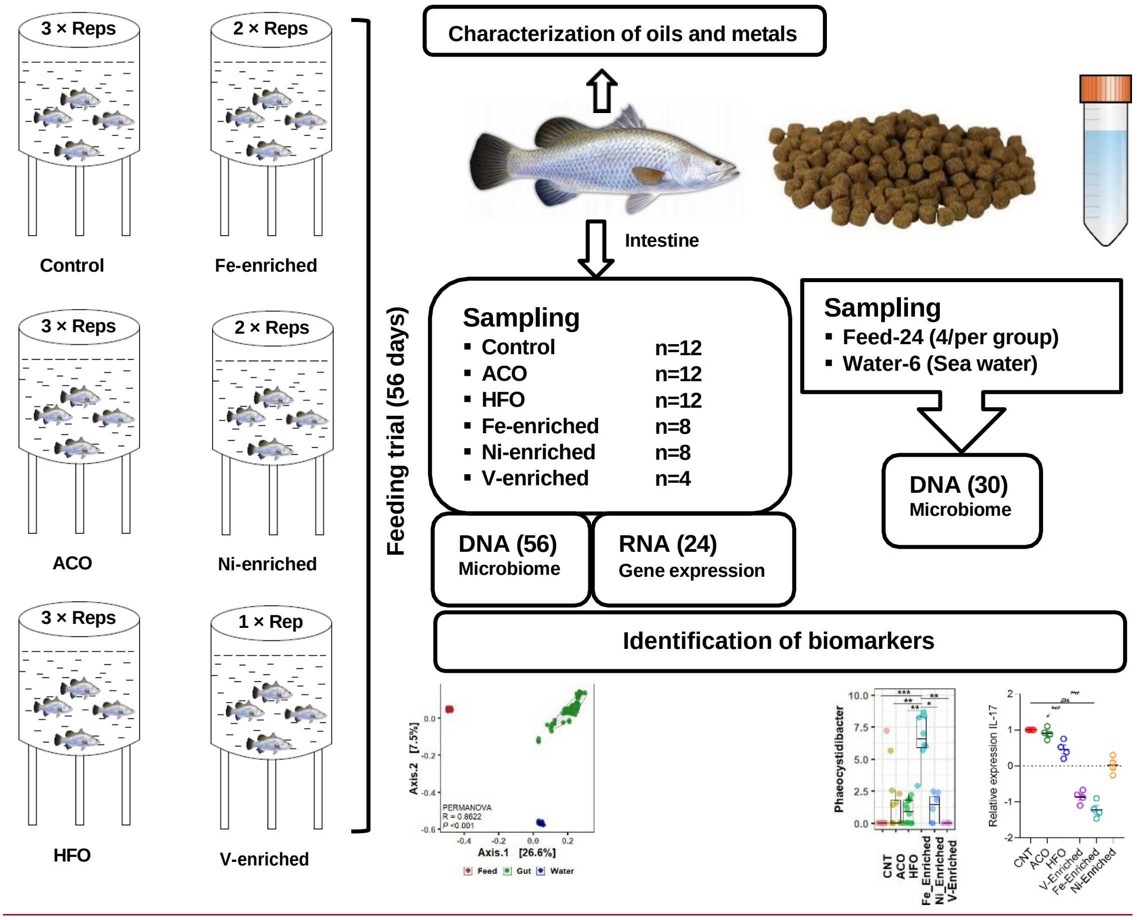

Figure S1. Flow-chart presenting the study design and sampling method

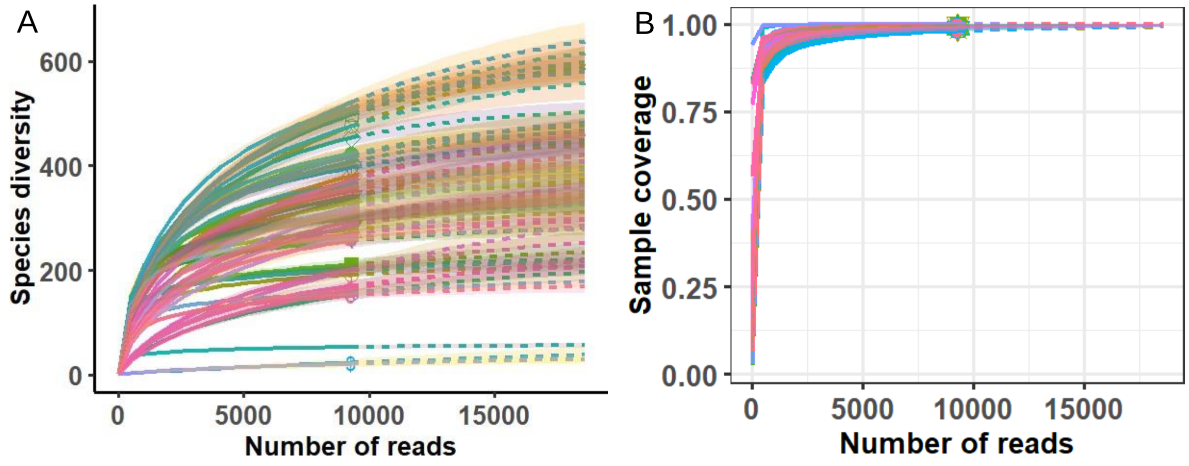

Figure S2. Rarefaction curve showing the (A) depth and (B) coverage of the sequences.

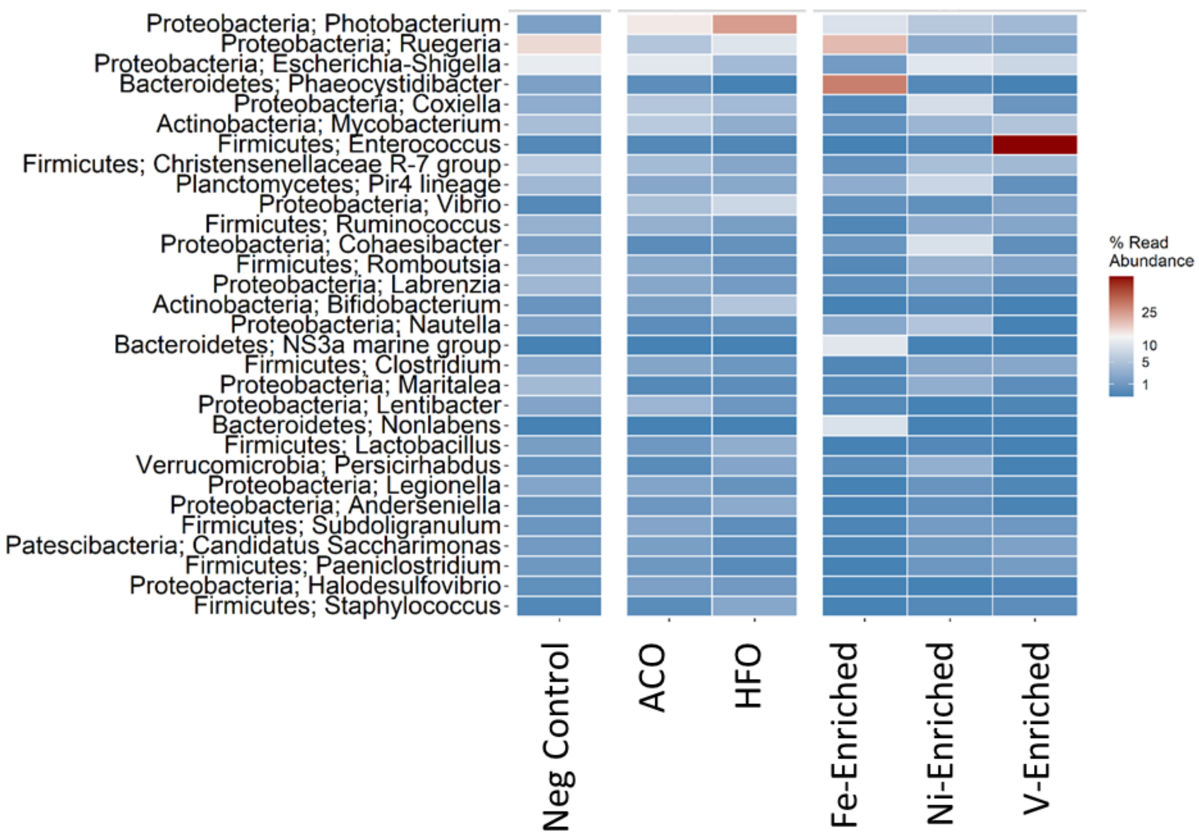

Figure S3. Relative abundance of bacteria at genus level in the gut of barramundi with six different diets.

Abbreviations: ACO, Australian Crude Oil; HFO, Heavy Fuel Oil.
